# 非小细胞肺癌原发灶与转移部位驱动基因状态一致性的研究进展

**DOI:** 10.3779/j.issn.1009-3419.2020.03.10

**Published:** 2020-03-20

**Authors:** 兵 周, 建萍 熊

**Affiliations:** 1 332000 九江，九江市第一人民医院/南昌大学附属九江医院病理科 Department of Pathology, The Affiliated Jiujiang Hospital of Nanchang University, Jiujiang 332000, China; 2 330006 南昌，南昌大学第一附属医院肿瘤内科 Department of Oncology, The First Affiliated Hospital of Nanchang University, Nanchang 330006, China

**Keywords:** 肺肿瘤, 驱动基因, 原发灶, 转移部位, Lung neoplasms, Driver gene, Primary lesions, Metastatic lesions

## Abstract

非小细胞肺癌（non-small cell lung cancer, NSCLC）作为肺癌最常见的病理类型，具有恶性程度高和侵袭性强的特点，晚期极易发生淋巴结和不同脏器转移。近年来，随着精准医学应用的深入，不断出现NSCLC转移部位的耐药和治疗失效为分子靶向治疗带来困扰，研究证实这些可能与肿瘤转移后发生的分子学改变有关。本文旨在阐述NSCLC原发灶与转移部位驱动基因状态，系统性综述两者间驱动基因状态一致性的研究进展，为探讨转移性NSCLC的分子靶向治疗提供新的思路。

## Abstract (en)

Non-small cell lung cancer (NSCLC), as the most common pathological type of lung cancer, has the characteristics of high malignancy and aggressiveness, and the lymph nodes and different organ metastases are prone to occur in advanced stages. In recent years, with the development of precision medicine, drug resistance and treatment failure in NSCLC metastasis sites have brought difficulties for molecular targeted therapy. And studies have confirmed that these may be related to new molecular changes after tumor metastasis. This review is to describe the state of the driving genes in the primary and metastatic sites of NSCLC, and systematically review the research progress on the consistency of the state of the driving genes between the primary and corresponding metastatic lesions, which provides a new idea for exploring the molecular targeted therapy of metastatic NSCLC.

随着恶性肿瘤分子学改变的发现和基因治疗学的不断发展，全面而准确的驱动基因检测结果对非小细胞肺癌（non-small cell lung cancer, NSCLC）的个体化治疗显得尤为重要^[1]^。然而，伴随着患者带瘤生存期的延长和分子靶向药物广泛应用，有研究^[2]^证实NSCLC转移后驱动基因状态发生了不同程度的改变，同时新的驱动基因突变产生的获得性耐药也给靶向治疗带来了新的难题。美国临床肿瘤学协会（American Society of Clinical Oncology, ASCO）2018年指南建议对晚期NSCLC患者，无论临床特征如何，均应对表皮生长因子受体（epidermal growth factor receptor, *EGFR*）、间变性淋巴瘤激酶（anaplastic lymphoma kinase, *ALK*）、鼠类肉瘤滤过性毒菌致癌同源体B1（v-raf murine sarcoma viral oncogene homolog B1, *BRAF*）和*ROS1*（c-ros oncogene 1 receptor tyrosine kinase）基因进行分子检测，而作为相对独立的预后因素，Kirsten鼠肉瘤病毒癌基因（Kirsten rat sarcoma viral oncogene homolog, *KRAS*）、肝细胞生长因子受体（hepatocyte growth factor receptor, HGF/c-MET)和人表皮生长因子受体2（human epidermal growth factor receptor 2, *HER2*）等基因也与肿瘤转移、治疗后耐药和评价预后有关，建议当前4种基因检测阴性或合并更大的组合时，后3种基因检测同样值得推荐；若同时肿瘤发生转移，应对原发灶和转移部位分别使用合适的分子检测基因靶点来指导治疗^[3]^。虽然之前已有少许NSCLC原发肿瘤和转移部位驱动基因状态的研究^[4, 5]^，但两者的突变数量和产生新的突变差异尚不清楚。本文拟就NSCLC原发灶与转移部位ASCO推荐的驱动基因状态一致性进行综述。

## 1 *EGFR*突变状态一致性

*EGFR*是目前NSCLC驱动基因中研究的最为透彻的分子靶点，由170 kDa跨膜糖肽构成，属于*c-erb-b1*原癌基因家族编码的受体酪氨酸激酶^[6]^。当信号分子激活蛋白激酶致EGFR胞内酪氨酸的残基自动磷酸化，激活下游多个的信号通路（RAS/RAF/MEK/MAPK、JAK/STAT及PI3K/PKB/mTOR），导致肿瘤细胞不断增殖、抑制凋亡和新血管生成，从而促使NSCLC的发生和转移^[7]^。

美国学者Schmid等^[8]^通过直接双向测序法对43例原发性肺腺癌和相应的淋巴结转移标本进行了*EGFR*基因18号-21号外显子检测，发现原发灶*EGFR*突变4例，其中3例为19号外显子缺失，1例21号外显子L858R突变；转移灶突变4例，其中外显子缺失仅1例，L858R突变2例，20号外显子插入突变发现1例，除了1例19号外显子缺失原发与转移灶一致外，其余突变病例均不相同。希腊学者Kalikaki等^[9]^应用直接测序法比较了25例NSCLC与相应转移灶中*EGFR*突变，原发灶5例突变中2例为19号外显子缺失，另外3例分别是18号外显子L692P、21号外显子G857E和E746V的罕见突变位点；转移部位突变3例，2例双重基因突变为19号外显子缺失+20号外显子S768i突变和L692P+V717A突变，另1例同样为罕见突变位点T847A，原发灶与转移部位*EGFR*突变状态不一致。韩国学者Gow等^[10]^采用直接测序法分析了67例有对应转移部位的NSCLC患者的*EGFR*突变，在原发灶突变阳性的18例中有9例转移部位发生突变，而在26例转移部位*EGFR*突变阳性者，原发肿瘤阳性仅9例，之间*EGFR*基因表达的不一致率达到27%（18/67），其中不一致的突变位点主要表现为野生型与以19号外显子缺失和21号外显子L858R为主的*EGFR*突变。国内学者方勤等^[11]^应用TaqMan RT-PCR的方法检测35例NSCLC原发灶和相应淋巴结转移的*EGFR*突变结果显示，其中18例原发灶和转移部位*EGFR*突变位点相同，11例原发灶发生突变，而转移部位为野生型，两者间*EGFR*基因表达不一致率达31.43%，不一致突变位点同样为19号外显子缺失和21号外显子L858R突变。值得注意的是，部分研究发现相对于NSCLC原发灶，其转移部位更容易出现20号插入子*EGFR*-T790M突变。波兰学者Powrózek等^[12]^采用实时荧光定量PCR分析143例NSCLC患者在脑转移的组织学标本中T790M突变，发现其特异性序列的扩增率增加到17.5%（25/143），远远超过原发灶报道的5.8%。美国学者Cai等^[13]^通过RT-PCR方法对比了11例肺腺癌原发灶和43例转移部位的*EGFR*突变，发现转移部位9例*EGFR*突变中出现了1例T790M突变，而原发灶5例中并未发现相关突变。韩国学者Han等^[14]^通过荧光定量PCR研究肺腺癌原发灶20例和相应转移部位19例基因突变情况，发现*EGFR*突变在两者之间的不一致率为20.8%（5/24），其中3例*EGFR*突变的原发灶病例在转移部位为野生型，2例转移部位*EGFR*突变而原发灶野生型病例中发现了1例T790M突变。

部分学者的研究明确到具体的转移器官，其中脑作为NSCLC转移最主要的靶器官，与原发灶的差异目前研究结果较为肯定，日本学者Matsumoto等^[15]^应用直接测序检出肺腺癌脑转移病灶*EGFR*突变率为63%（12/19），明显高于同期原发灶的20%-55%的突变频率。国内学者王帅等^[16]^应用直接测序及突变扩增系统（amplification refractory mutation system, ARMS）检测31例肺原发灶和脑转移灶*EGFR*突变状态，发现肺原发灶和脑转移灶*EGFR*突变状况不一致为16.1%（5/31），不一致突变位点为原发灶*EGFR*野生型而脑转移灶检出19号外显子缺失和21号外显子L858R突变。台湾学者Rau等^[17]^在通过ARMS法检测49例肺腺癌原发灶和对应脑转移中*EGFR*突变状态发现表达不一致率为26.5%（13/49），脑转移灶不一致突变位点以19号外显子缺失突变为主，而原发灶未检出。然而，在NSCLC除了脑外其他器官转移的*EGFR*突变一致性还存在分歧，日本学者Takano、Yasunorii与Fujimoto等^[18-20]^同时发现，当肺腺癌发生了骨、脑或者肝转移后，*EGFR*基因突变病例较EGFR野生型病例转移时病灶数目更多，且转移器官中更容易被检测到*EGFR* 19号外显子缺失和21号外显子L858R点突变。荷兰学者Smits等^[21]^通过ARMS法和直接测序法对261例肺腺癌原发灶和转移部位的*EGFR*基因突变率进行检测，发现相对于原发灶11.4%的突变率，脑转移患者*EGFR*突变频率上升到25%，心包转移增加到26.5%，而对比其他转移部位（如肝、骨及软组织）EGFR并未发现明显差异。

然而，也有部分国内学者认为NSCLC的原发灶与转移部位的EGFR改变并无明显差异。Wei等^[22]^采用荧光定量PCR检测NSCLC原发灶与转移部位的*EGFR*突变率，发现原发灶*EGFR*突变50例中，19号外显子缺失28例，21号外显子L858R突变22例；转移部位突变47例，19号外显子缺失和21号外显子L858R突变分别为26例和21例，所有对应的原发灶和转移部位中表现出相同的突变位点，证实两者存在高度的一致性。李剑英等^[23]^通过收集原发性肺腺癌182例和相应转移瘤109例，研究发现转移灶与原发灶*EGFR*基因突变率分别为58.2%和56.9%，总体一致率较好，能够通过转移灶预测原发灶的基因学变化。马洁韬等^[24]^比较配对的NSCLC原发灶和转移淋巴结*EGFR*基因状态，发现原发灶和与对应的淋巴结转移中*EGFR*基因突变率分别为31.82%（7/22）和27.27%（6/22），*EGFR*基因型一致率达95.45%，且两组突变位点相同，证实肺癌原发灶和相应转移淋巴结中*EGFR*基因突变较稳定。

总体来说，多数研究均表明NSCLC发生转移时*EGFR*基因状态存在不稳定性，即出现转移部位与原发肿瘤的基因状态不一致的情况，而脑作为最常见的转移部位，*EGFR*突变频率出现明显的升高。同时我们还注意到除了由于检测手段改进和试剂盒探针检测范围的增加出现罕见外显子突变位点外，国外学者在肺腺癌转移灶中发现了20号插入子T790M的继发性*EGFR*基因突变，虽然总体突变率不高，但对于晚期NSCLC重新制定治疗策略与评估预后有极大的临床意义。

## 2 *ALK*突变状态一致性

*ALK*作为新发现的NSCLC驱动基因，虽然不像EGFR研究透彻，但越来越受重视^[25]^。其属于胰岛素受体家族的一类跨膜受体酪氨酸激酶蛋白，当它与棘皮动物微管相关蛋白（echinoderm microtubule associated protein-like 4, EML4）片段发生倒位融合时，能够表达新的*EML4-ALK*融合蛋白，从而导致ALK的胞内激酶结构的二聚化和下游激酶信号转导，通过多个信号通路（PI3K/AKT/mTOR、MAPK和JAK/STAT），导致肿瘤细胞不断增殖、抑制细胞凋亡，从而促使NSCLC的发生侵袭和转移^[26]^。

目前不断有研究发现携带*ALK*基因突变的NSCLC与更积极的临床行为有关。美国学者Cai等^[13]^通过荧光原位杂交（fluorescence *in situ* hybridization, FISH）检测的22例不表达*EGFR*和*KRAS*的肺腺癌转移部位中发现3例*ALK*基因重排，而在原发灶中则未检出，证实转移灶中*ALK*重排更加常见。意大利学者Rossi等^[27]^报道了1例54岁肺腺癌患者*ALK*重排的差异，该患者原发灶*ALK*重排阴性，8个月后转移性腹膜活检标本FISH检测发现*ALK*融合基因。美国学者Doebele等^[28]^通过FISH检测研究209例NSCLC原发灶与对应心包转移灶与*ALK*重排的关系，发现原发灶中*ALK*重排率为9%（18/209），而发生了心包转移后*ALK*重排率达到20%（41/209）。韩国学者Kim等^[29]^采用FISH和免疫组织化学染色（immunohistochemistry, IHC）方法检测67例NSCLC原发灶及其相应转移部位ALK的表达情况，得出*ALK*重排仅见于肺腺癌，原发肿瘤中的ALK在表达率为11.9%（8/67），而在转移部位中的表达率上升到25.4%（17/67），显示ALK的表达伴随着肿瘤的转移而增加。但也有个别学者认为ALK的表达在两者存在不一致且与转移存在负相关，意大利学者Bozzetti等^[30]^通过FISH法评估NSCLC原发与转移灶直接细胞学涂片的*ALK*重排情况，发现原发灶*ALK*重排率为23%（3/13），而转移部位重排率为19%（7/36），转移灶相对较低。国内学者王琳等^[31]^在通过荧光定量PCR法检测多中心配对的246例NSCLC原发灶和转移部位中*ALK*融合基因，发现配对原发灶阳性率11.0%（27/246），而配对转移灶为7.3%（18/246），其中转移灶阳性对应的原发灶表达阴性2例，而原发灶阳性对应的转移灶阴性11例，原发灶较转移灶检出*ALK*融合基因阳性率高。

同时也有部分学者认为NSCLC原发灶及其相应转移部位的*ALK*基因突变一致性较好，美国学者Bittar等^[32]^通过FISH与IHC法分析对比68例原发肺肿瘤和转移部位的ALK表达，发现利用FISH检测原发部位和转移部位*ALK*重排的一致性为88%，IHC检测两者ALK重排一致性为94%，两者共5例不一致，其中3例FISH阴性而IHC弱阳性，2例FISH检测到重排而IHC结果阴性；FISH和IHC的方法检测ALK结果总体一致性为88%。国内学者Hou等^[33]^采用IHC、RT-PCR及FISH检测101例NSCLC原发灶及转移部位*ALK*突变发现，其中原发灶阳性率为20%（20/101），而转移灶阳性率为18%（18/101），两者的一致性达98%，且同一患者转移淋巴结的不同部位的*ALK*重排是一致的。国内学者Ma等^[34]^通过收集了106例ALK阳性肺腺癌病例，通过Ventana全自动免疫组化染色分析其中有53例淋巴结或其他部位转移灶的*ALK*基因表达情况，在原发肿瘤与相应的淋巴结转移之间没有发现ALK表达不一致的情况。

综上，目前对于原发灶与转移灶*ALK*基因检测结果的一致性还存在一定的争议，但相对来说认为*ALK*基因原发灶与转移部位间发生改变占多数。临床研究证实特异性靶向激酶抑制剂，如ALK-酪氨酸激酶抑制剂（tyrosine kinase inhibitor, TKIs），极大地改善了ALK阳性的NSCLC的治疗策略，能够显著地延长生存期，因此精准的*ALK*基因检测结果意义重大。选择合适的检测手段、转移灶多部位取材检测和更多的肿瘤样本含量能够克服肿瘤病灶产生的异质性，增加ALK检测的敏感性。

## 3 *KRAS*突变状态一致性

*KRAS*是定位于*EGFR*基因信号通路下游的12号染色体的原癌基因^[35]^。其在信号转导通路中作用巨大，有“分子开关”之称，正常的*KRAS*基因能够与GTP结合，切掉GTP的磷酸基生成GDP从而抑制肿瘤的生成，当发生*KRAS*基因活化突变时，GTP不能去掉磷酸基而保存GTP的活化状态，会促进肿瘤细胞的增殖和间质血管的生成而导致肿瘤的发生^[36]^。*KRAS*基因与NSCLC发生关系密切，当发生*KRAS*基因突变时，往往预示着针对EGFR的靶向药物耐药，且整体预后较KRAS野生型差^[37]^。

目前不断有学者证实NSCLC原发灶与转移部位的*KRAS*基因突变不一致，日本学者Maemondo等^[38]^比较了40例肺癌原发瘤与转移部位样本中*KRAS*的基因状态，发现其中出现了9例（22.5%）的表达不同。台湾学者Rau等^[17]^在通过一代测序和ARMS分析*KRAS*在原发肿瘤和匹配脑转移中的不一致突变状态发现，49例原发肺腺癌和对应的脑转移灶中，33例患者配对标本中可检出*KRAS*基因，不一致率达39.4%（13/33）。希腊学者Kalikaki等^[9]^比较了25例原发性肿瘤与相应转移灶中这些基因的突变情况发现，原发性肿瘤与相应转移癌的和*KRAS*突变情况不一致率为24%，在5例原发肿瘤*KRAS*突变患者中，只有2例存在相同的转移突变。同时也有学者指出在*KRAS*基因状态不一致同时，原发灶突变率要高于转移部位。荷兰学者Smits等^[21]^通过ARMS法和直接测序法对261例肺腺癌原发灶和转移部位的*KRAS*基因突变率进行检测，发现相对于原发灶40.1%的突变率，转移部位总体突变频率下降到31.3%，其中肝转移下降到22.7%，心包转移下降到18.8%，脑转移下降到9.1%。美国学者Katharina等^[8]^通过直接双向测序法对43例原发性肺腺癌和相应的淋巴结转移标本进行了*KRAS*基因密码子12、13和61-68检测，原发灶突变率为60.9%（28/46），淋巴结转移灶突变率为43.5%（20/46），且其中有25例在原发肿瘤和相应的淋巴结转移中发现不一致的突变状态。国内学者韩琤波等^[39]^通过大样本*meta*分析193例NSCLC原发和转移灶发现，原发灶*KRAS*突变50例（25.91%），而在转移灶中仅突变36例（18.65%），原发灶中*KRAS*基因突变频率高于转移灶，认为转移灶更能反映肺癌周身癌灶*KRAS*基因特征，单独对转移灶做*KRAS*状态分析可能会引入更多抵抗EGFR-TKI治疗的患者。Rodriguez等^[40]^通过二代测序方法研究18例转移灶和15例转移灶中*KRAS*表达发现，*KRAS*在转移瘤中发生3例突变，而在原发肿瘤中有4例表达，证实转移瘤*KRAS*突变的频率低于原发性NSCLC。但也有少数学者认为*KRAS*基因突变率转移部位要高于原发灶。美国学者Cai等^[13]^通过对11例NSCLC和43例对应的转移性瘤行PCR检测*KRAS*突变发现，在检测的14例标本中有3例（21%）出现*KRAS*突变，其中1例原发灶和2例转移瘤，显示转移瘤多1例。国内学者孙雷娜等^[41]^收集80例NSCLC病例标本，利用直接测序和实时荧光定量PCR的方法分别检侧原发灶和相应淋巴结转移灶中*KRAS*基因第12、13密码子的突变情况，发现80例NSCLC患者中，检出原发灶携带*KRAS*突变为1例，而检出转移灶携带*KRAS*基因突变7例，证实转移灶中*KRAS*突变更常见。

然而也有部分学者持不同意见，认为晚期NSCLC原发灶与转移部位*KRAS*基因突变无明显的不一致性。英国学者Delicia等^[42]^通过ARMS法研究*KRAS*在60例转移性肺腺癌中的突变发现，相对于在原发性肺腺癌中*KRAS*的突变率26.7%（16/60），转移瘤突变率为28.3%（17/60），转移部位仅发生了1例获得性突变，无统计学差异。国内学者马洁韬等^[24]^通过直接测序比较22例原发与转移的*KRAS*发现基因突变率分别为2/22和1/22，没有统计学差异，暗示在某些情况下，*KRAS*突变可能会导致肿瘤细胞的增殖，但与发生转移并无关系。

总之，目前认为NSCLC原发肿瘤与转移部位的*KRAS*基因状态不一致的情况还是占了大多数，且伴随着肿瘤的转移*KRAS*基因的突变率会发生降低。但由于纳入研究以国外文献为主，且相对于欧美NSCLC的30% *KRAS*基因突变率，亚洲人群突变率不足10%，是否会影响一致性结果的判断还待进一步研究。当发生*KRAS*基因突变时，往往预示着针对EGFR的靶向药物耐药，且整体预后较*KRAS*野生型差，排除技术和标本肿瘤含量等关系，重复检测具有实际的意义。

## 4 *c-MET*突变状态一致性

c-MET由50 kDa的α亚基和140 kDa的β亚基组成，属于原癌基因酪氨酸激酶受体（receptor tyrosine kinase, RTK）家族一员，其胞外区可结合HGF通过酪氨酸残基磷酸化激活下游信号通路，导致多种生物活性如促进肿瘤细胞增殖、生长、侵袭、抑制凋亡和血管生成及上皮-间充质转化（epithelial to mesenchymal transition, EMT）等^[43]^。*c-MET*基因的扩增被认为与NSCLC EGFR靶向耐药关系密切，能通过激活*EGFR*非依赖的HER3、MAPK/ERK1/2T及ERBB3/PI3K/AKT等信号通路促进下游信号转导诱导肿瘤细胞对EGFR-TKIs的耐药，因此对于原发和继发NSCLC的*c-MET*基因检测就显得尤为重要^[44]^。

澳大利亚学者Tran等^[45]^通过IHC及FISH法检测300例NSCLC原发灶以及对应的93例转移灶的*c-MET*基因状态，原发灶IHC法和FISH法检测阳性率分别为9.3%（28/300）和8.1%（22/300），而在转移灶中阳性率分别高达18.3%（17/93）和21.3%（20/93）；同时还发现鳞状细胞癌相比腺癌c-MET过表达较低，c-MET阳性病例的总体生存率都有明显的提高。国内学者Xu等^[46]^采用实时荧光定量PCR技术检测178例原发灶及配对转移淋巴结标本的*c-MET*基因扩增状态，发现原发灶中*c-MET*基因扩增阳性率为7.95%（15/178），而转移淋巴结中*c-MET*基因扩增阳性率为达18.54%（33/178），证实*c-MET*基因扩增在两者间存在明显不一致，转移性淋巴结中的检出率高于在原发性癌组织中的检出率；以转移淋巴结作为原发性癌组织的代用品，敏感性为86.67%（13/15），特异性为87.69%（143/163）。国内学者Han等^[47]^通过荧光定量PCR研究22例NSCLC患者及其淋巴结转移灶的*c-MET*扩增率发现，转移灶中*c-MET*基因拷贝数（gene copy number, GCN）明显高于原发灶且存在统计学意义，证实c-MET GCN在EGFR-TKI抵抗和淋巴结转移性肿瘤患者中明显升高。李扬等^[48]^通过RT-PCR法研究晚期NSCLC原发灶和转移灶的*c-MET*扩增阳性率发现，在147例原发灶和71例转移灶中，原发灶阳性率为8.84%（13/147），而转移灶中阳性率为18.31%（13/71），证实*c-MET*扩增转移灶阳性率高于原发灶，检测转移灶能筛出更多有适应证的患者。马洁韬等^[24]^通过检查22例原发瘤与转移灶发现，转移灶*c-MET*基因拷贝数显著高于原发灶。这可能与EGFR-TKI治疗后*MET*基因扩增相关获得性耐药与EGFR-TKI治疗后*MET*基因得到进一步选择积聚有关。

虽然目前对于NSCLC原发灶与转移部位的*c-MET*基因突变一致性的研究较少，但得到的结果都较为积极，即不仅存在一致性的差异，同时发生转移后*c-MET*基因的突变率明显增高。已有研究表明分子靶向药物克唑替尼在*c-MET*基因扩增患者中取得了良好的疗效，可通过阻止EGFR信号通路传导和酪氨酸残基磷酸化来抑制靶向治疗的耐药性。NSCLC转移部位*c-MET*基因的重复检测对于临床制定治疗策略有极大的意义。

## 5 其他基因突变状态一致性

除外以上研究较多的驱动基因，NSCLC原发瘤与转移部位驱动基因*ROS1*、*Braf*、*Her2*、*TP53*（tumor protein p53）等也有少量配对研究，一致性也存在着同样的争议。目前意大利学者Bozzetti等^[30]^通过FISH法评估NSCLC原发与转移灶直接细胞学涂片的*ROS1*扩增情况，发现原发灶2例有1例扩增，而转移部位10例有2例扩增，虽然病例量较少，但预示ROS1在两者之间存在着一定的不同。王琳等^[49]^通过荧光定量PCR法多中心探讨肺癌原发灶和转移灶ROS1融合基因阳性率相关性，发现在384例原发灶和246例配对转移灶中，ROS1融合基因阳性率原发灶为2.60%（10/384），配对原发灶为2.85%（7/246），配对转移灶为1.63%（4/246），配对的246对原发灶、转移灶组织中，转移灶融合基因阳性而对应的原发灶融合基因阴性1例，原发灶融合基因阳性而对应的转移灶融合基因阴性4例，原发灶较转移灶检出ROS1融合基因阳性率高。刘晓辉等^[50]^也得出了同样的结论，他们通过实时荧光定量PCR研究NSCLC原发灶162例和配对转移灶139例发现，晚期NSCLC原发灶及配对转移灶*ROS1*融合基因表达阳性率分别为4.3%（7/162）和2.2%（3/139），证实原发灶较转移灶ROS1融合基因检出阳性率显著增高。但两项研究同时指出配对原发灶及转移灶的*ROS1*融合基因与性别、年龄、吸烟情况和病理类型相关性均不显著。澳大利亚学者Katharina等^[8]^通过双向测序法对43例原发性肺腺癌和相应的肺局部淋巴结转移标本进行了BARF第15号外显子检测，在2例原发性肺腺癌病例中分别发现了1例框架内缺失和1例K601L错义点突变，而所有淋巴结转移病例均为野生型BRAF，未发现突变病例。意大利学者Fassan等^[51]^通过IHC与FISH方法研究35例原发和对应的转移瘤的HER2状态的差异，发现两者之间总体一致性较好，仅有1例患者有不同的评分（2分*vs* 3分），但之间的差异对临床无影响。国内学者Wu等^[52]^对35例原发性肺腺癌和35例相应的淋巴结转移进行了基因组和转录的一致性分析，发现*TP53*突变在转移癌患者的肿瘤中明显富集，调节细胞骨架重塑过程的基因也经常被改变，尤其是在转移的样本中，证实突变体TP53不仅失去了抑瘤活性，而且还与促进肿瘤不同肿瘤类型的侵袭、迁移和转移有关。

## 6 小结与展望

本文总结了既往NSCLC原发灶与转移部位各驱动因子的一致性研究，大部分学者认为原发灶与转移部位驱动基因状态存在着明显的不一致性。有研究^[53]^通过细胞系标记证实肿瘤的转移所产生的不一致性并不是一个随机的过程，而是经过了基因的程序化控制的结果。同时也有研究^[54]^指出驱动基因的不一致性可能与肿瘤细胞不同的克隆、肿瘤遗传的异质性、转移部位的获得性、检测方法敏感性及肿瘤细胞含量有关。因此，我们日常工作中在应对NSCLC转移部位驱动基因的评估时，应该努力克服标本肿瘤含量和检测技术造成的差异，注重转移部位的多灶取材和尽可能多地送检肿瘤组织从而增加检测敏感性，同时在驱动基因检测技术上遵循2018年ASCO指南建议各驱动基因的检测手段加以检测^[3]^。当然，排除技术和标本肿瘤含量等关系，肿瘤的异质性和继发性耐药无法避免，转移部位重复的基因检测具有实际的意义。由于本次纳入文献较少，期待未来更多大样本和多中心的前瞻性研究，进一步明确各驱动因子在肺癌原发灶和转移部位的差别，为晚期肺癌发展和转移后临床重新制定治疗方案打下坚实的基础。
